# Effect of Graphene Nanoplatelets (GNPs) on Fatigue Properties of Asphalt Mastics

**DOI:** 10.3390/ma14174864

**Published:** 2021-08-27

**Authors:** Ke Li, Haisheng Ren, Weirong Huang

**Affiliations:** 1 Sichuan Polytechnic Technician College, Chengdu 611130, China; runninglike@163.com; 2 Intelligent Transportation System Research Center, School of Transportation, Southeast University, Nanjing 210096, China; 3 School of Materials Science and Engineering, Chongqing Jiaotong University, Chongqing 400074, China

**Keywords:** graphene, asphalt mastics, filler-asphalt ratio, fatigue properties

## Abstract

To investigate the effect of graphene on the fatigue properties of base asphalt mastics, graphene nanoplatelets (GNPs)-modified asphalt mastics and base asphalt mastics were prepared. A dynamic shear rheometer (DSR) was used to conduct the tests in the stress-controlled mode of a time-sweep test. The results showed that GNPs can improve the fatigue life of asphalt mastic. Under a stress of 0.15 MPa, the average fatigue life growth rate ($\bar{\omega }$) was 17.7% at a filler-asphalt ratio of 0.8, 35.4% at 1.0, and 45.2% at 1.2; under a stress of 0.2 MPa, the average fatigue life growth rate ($\bar{\omega }$) was 17.9% at a filler-asphalt ratio of 0.8, 25.6% at 1.0, and 38.2% at 1.2. The growth value (ΔT) of fatigue life of GNPs-modified asphalt mastics increased correspondingly with the increase of filler–asphalt ratio, the correlation coefficient R^2^ was greater than 0.95, and the growth amount showed a good linear relationship with the filler–asphalt ratio. In the range of 0.8~1.2 filler–asphalt ratio, the increase of mineral powder can improve the fatigue life of asphalt mastics, and there is a good linear correlation between filler–asphalt ratio and fatigue life. The anti-fatigue mechanism of GNPs lies in the interaction between GNPs and asphalt, as well as its own lubricity and thermal conductivity.

## 1. Introduction

Asphalt pavements will be subjected to repeated loads of vehicles during use, which makes fatigue damage one of the main forms of pavement diseases. As the main binder of the pavement material, the fatigue failure of the pavement is mainly attributed to the fatigue cracking of asphalt. First of all, under the initial load, the micro-cracking of asphalt gradually expands to macro-cracking with the continuous application of load, which makes the interface between aggregates break each other, and finally shows the pavement cracking [1,2].

The research on graphene-modified asphalt conducted by road materials researchers based on the excellent properties of graphene material itself has made some progress, but it is still at a relatively early stage. Marasteanu et al. [3] studied the influence of graphene addition on the compaction performance of asphalt mixture. Research shows that graphene can be used as a “lubricant”, which can effectively reduce the compression force of the asphalt mixture. Without graphene, it takes about 60 revolutions to compact the mixed porosity to 5%, while after adding graphene at 28% of the asphalt mass, the number of resolution is only 20. Zhou et al. [4] studied the influence of graphene on the thermodynamic properties of asphalt by molecular simulation and experimental comparison, and studied its thermal stability and glass transition temperature by differential scanning calorimetry; The results show that the glass transition temperature Tg of the graphene-modified asphalt changes by 251 K and 276 K, respectively, for asphalt and modified asphalt. Indicating that graphene can improve the Tg and thermal properties. Moreno-Navarro et al. [5] conducted heat conduction on asphalt and graphene modified asphalt samples, and measured that the time required for the samples to rise to 5 °C was 182 s and 101 s, respectively, indicating that graphene can improve the thermal conductivity of materials. To solve the problems of poor compatibility and low temperature performance of graphene-modified asphalt, Han et al. [6] prepared graphene (GNPs) grafted polystyrene (Ps) composite by in situ polymerization, and obtained modified SBS, which was compounded with matrix asphalt to obtain PS-GNPs/SBS modified asphalt. The results show that the addition of PS-GNPs can effectively improve the compatibility, plasticity, viscoelasticity, rutting resistance at high temperature, fatigue resistance and low temperature performance of the material. Han et al. [7] addressed the problem of poor compatibility affecting the modification effect of graphene (GNPs) incorporated into SBS-modified asphalt by using a covalent bonding method to graft octadecylamine (ODA) onto the surface of GNPs to obtain ODA-GNPs complexes. ODA-GNPs and SBS modifier were used to prepare ODA-GNPs/SBS modified asphalt. The results show that ODA grafting enhances the lipophilicity of GNPs in asphalt, which leads to better dispersion effect. This further effectively improves the plasticity, high and low temperature performance and viscosity of the base asphalt. Li et al. [8] compared the performance of PS-GNPs/SBS modified asphalt with that of ODA-GNPs/SBS modified asphalt. Through a dynamic shear rheometer (DSR), multiple stress creep recovery test (MSCR), bending beam rheometer (BBR), time scanning, Marshall water stability, freeze-thaw splitting and a rutting test, it was shown that the mechanical properties and water damage resistance of PS-GNPs/SBS modified asphalt were better than those of ODA-GNPs/SBS modified asphalt. The fatigue life N_f50_ of PS-GNPs/SBS modified asphalt with 0.02% GNPs content was 272.79% higher than that of the original SBS asphalt; the fatigue life N_f50_ of ODA-GNPs/SBS modified asphalt with 0.08% GNPs content was 247.19% higher than that of the original SBS asphalt. Su et al. [9] prepared aminated graphene (NH 2-GNPs/D-PAN) fiber modified asphalt by using dopamine self-polymerization and aminated graphene covalent grafting to modify polyacrylonitrile (PAN) fibers. It was shown that NH2-GNPs/D-PAN asphalt had better viscoelasticity, permanent deformation resistance, low-temperature crack resistance and water damage properties than the original fiber asphalt. Guo et al. [10] improved the performance of asphalt through the synergistic enhancement of graphene and tourmaline. Graphene/tourmaline composite-modified asphalt was prepared, and its rheological properties are mainly studied. The results showed that the rutting resistance of graphene/tourmaline composite-modified asphalt was much higher than that of tourmaline-modified asphalt. Li et al. [11] used polymethylmethacrylate (PMMA) and GNPs to prepare composite PMMA-GNPs by microwave heating emulsion polymerization, and then prepared PMMA-GNPs/SBS modified asphalt together with SBS. The results showed that microwave heating could significantly shorten the reaction time compared with traditional water bath heating or oil bath heating. Compared with SBS modified asphalt, the addition of PMMA-GNPs could enhance the rutting resistance, reduce the sensitivity to stress changes and improve the storage stability at high temperature. Chen et al. [12] prepared GNPs/rubber powder composite modified asphalt with graphene (GNP_S_) as a modifier in order to improve the compatibility of rubber powder-modified asphalt. The results showed that the addition of graphene could improve the compatibility and adhesion of rubber powder-modified asphalt. As well as improving the high and low temperature properties and viscoelasticity of rubber powder-modified asphalt. It was proposed that GNPs/rubber powder composite modified asphalt is suitable for heavy-duty traffic. Huang et al. [13] used dipropylene glycol dimethyl ether (DME) and polyvinylpyrrolidone (PVP) as dispersants to prepare graphene mother liquor (GML). Based on the Lambert–Beer Law, the amount of graphene (G) and the ratio of dispersant to graphene (D/G) were obtained. GML and asphalt were compounded to prepare graphene modified asphalt DME-GMA and PVP-GMA respectively. Studies have shown that the dispersant can improve the dispersion effect of graphene in the base asphalt, and the dispersion effect of PVP is better than that of DME. Li et al. [14] used commercial graphene as a modifier without any treatment, and prepared different amounts of graphene modified asphalt. Its physical properties, anti-aging properties and rheological properties were analyzed. It was shown that the addition of graphene improved the high temperature performance of the material, and the graphene flakes were able to hinder and prevent the diffusion of oxygen into the asphalt, which resulted in excellent anti-aging properties. The modification mechanism and anti-aging mechanism were studied by micro-characterization, atomic force microscopy (AFM) and Fourier transform infrared spectroscopy (FTIR). It was shown that no new absorption peaks appeared in the FTIR spectra of the graphene-modified asphalt, indicating that graphene is physically dispersed in the asphalt and does not react chemically.

In a review of related graphene modified asphalt, Wu et al. [15] summarized the influence of graphene on asphalt binder from three aspects: rutting resistance (high temperature performance), fatigue cracking resistance (medium temperature performance) and thermal cracking resistance (low temperature performance). It was proposed that future research focus of graphene modified asphalt should be on fatigue cracking resistance and thermal cracking resistance. Han et al. [16] pointed out that the performance tests of graphene-modified asphalt mainly include viscoelasticity, high and low temperature stability, compatibility, electrical conductivity, self-healing and aging resistance; The performance tests of asphalt mixtures mainly include volume index and rutting resistance.

To sum up, some progress has been made in the field of graphene-modified asphalt. These studies focuses on the high and low temperature performance, aging performance and compatibility of asphalt. Studies have shown that graphene combines with asphalt as a physical action [14], and a large number of studies have been devoted to the problem of graphene–asphalt compatibility, where many properties of asphalt are improved under the premise of better compatibility. As for fatigue performance, a few studies have confirmed an improvement of the fatigue life of asphalt [11], combined with the lubricating properties of graphene [3] and the ability to increase the thermal conductivity of asphalt [4,5]. Based on these conclusions, this study has a certain theoretical basis. In the existing research, there are few reports on asphalt and asphalt mixture, but in the design and application of actual asphalt pavements, asphalt and mineral powder are combined into asphalt mastics, which acts as a filler and binder between the aggregates. Moreover, the existing research on the fatigue properties of graphene modified asphalt mostly focus on the fatigue properties of asphalt, and few research works on the fatigue properties of asphalt mastics. Therefore, this paper takes the asphalt mastics which are closer to practical application as the research object. The fatigue properties of basic asphalt mastics and GNPs-modified asphalt mastics were studied, and the changes of fatigue life and the influence of graphene on the fatigue life of asphalt mastics were compared. This paper enriches the study of graphene-modified asphalt.

## 2. Materials and Methods

### 2.1. Materials

In this paper, No. 70 Grade A asphalt was used, the performance indexes of which are shown in Table 1, and all tests were conducted in accordance with JTG E20-2011 [17] standard. In view of the agglomeration phenomenon of nanomaterials when they are compounded with asphalt, the surface-modified lipophilic graphene was prepared by the mechanical stripping method provided by a certain graphene technology company. The main performance indicators are shown in Table 2. The mineral powder was made from finely ground limestone, whose performance indexes are shown in Table 3, and all tests were conducted in accordance with the JTG E42-2005 [18] standard.

### 2.2. Sample Preparation

In our team’s previous research, the best doping amount of graphene in base asphalt was obtained. The recommended doping amount of graphene is 0.35% of asphalt mass, which will be directly adopted in this study.

According to the preparation principle of the melt mixing method [19,20], the amount of GNPs added is 0.35% of the asphalt mass. Firstly, the asphalt is heated to a liquid state, then graphene is added and stirred uniformly, and then the GNPs modified asphalt is prepared by a high-speed shearing machine. The preparation process parameters are as follows: 4500 r/min high-speed shear for 30 min, and the temperature is controlled at 135 °C to 145 °C with electric heating device. Then we slowly added mineral powder with filler-asphalt ratio of 0.8, 1.0 and 1.2, and fully mixed this for 10 min, thus preparing graphene-modified asphalt mastics. The base asphalt mastics prepared with the same filler-asphalt ratio was used as the control group.

### 2.3. Experimental Methods

#### 2.3.1. Fatigue Test

The NCHRP 9-10 project team in the United States using dynamic shear (DSR) time -sweep tests of asphalt under repeated shear loads to measure the fatigue performance of asphalt [1].

An American TA-DHR 2 dynamic shear rheometer (New Castle, DE, USA) was used as the testing instrument. The selection of filler-asphalt ratio is in accordance with the suggestion of the Technical Specification for Construction of Highway Asphalt Pavement (JTG F40-2004) [21]. The filler-asphalt ratio of common asphalt mixture should be controlled within the range of 0.8~1.2, and the base asphalt mastics and graphene-modified asphalt mastics with filler–asphalt ratio of 0.8, 1.0 and 1.2 were prepared. This was used to study the effect of mineral powder content on the fatigue properties of asphalt and the effect of the addition of graphene on the fatigue properties of asphalt mastics. The test samples are shown in Table 4.

#### 2.3.2. Fatigue Evaluation Index

In the time-sweep mode using the DSR, the fatigue properties of asphalt were evaluated as follows:

(1) N_f50_: when the initial modulus ($G_{0}^{*}$) decreases to 50%, it was judged as fatigue damage.
(2) N_δ_: the inflection point of the phase angle (δ) curve was defined as fatigue damage [22,23,24,25].

The evaluation indexes of N_f50_ and N_δ_ can be obtained directly from the experimental results, which is relatively easy [8].

**Figure 1:**
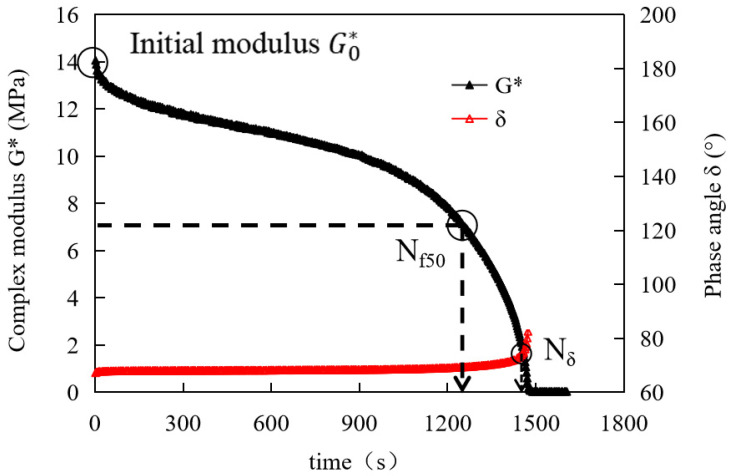
Asphalt mastics complex modulus (phase angle)—cyclic loading time curve.

The fatigue curve of typical asphalt mastic is shown in Figure 1. The evaluation index N_f50_ is defined as the loading time (T) corresponding to when the initial complex modulus ($G_{0}^{*}$) decreases to 50% is defined as the fatigue life of asphalt mastics (s); the evaluation index N_δ_ is defined as when the phase angle δ has an inflection point in the latter section, and the loading time (T) corresponding to the inflection point is defined as the fatigue life of asphalt mastics (s). After that, the data points are obviously dispersed, which is no longer desirable.

#### 2.3.3. Test Parameters

The loading control mode is the stress control mode [26], and the detailed test parameters are shown in Table 5.

## 3. Results and Discussion

### 3.1. Fatigue Life of Asphalt Mastics

Under the stress control mode of 0.15 MPa and 0.2 MPa, fatigue tests were carried out on base asphalt mastics and GNPs-modified asphalt mastics with filling asphalt ratios of 0.8, 1.0 and 1.2, respectively; The fatigue life (T) of each asphalt mastics was listed in Table 6 by evaluating the results of the index N_f50_ and N_δ_ collation tests. In order to make the results representative, the average value of the samples was taken as a representative value.

#### 3.1.1. Effect of Filler–Asphalt Ratio on Fatigue Properties of Asphalt Mastics

As for the influence of the change of filler-asphalt ratio on the fatigue life of asphalt mastics, the results from Table 6 show that the fatigue life of two kinds of asphalt mastics increases with the increase of filler–asphalt ratio under two evaluation indexes and two stress modes. This indicates that in the range of 0.8 to 1.2 filler–asphalt ratio, the increase of mineral powder has an improving effect on the fatigue life of asphalt mastics.

**Figure 2:**
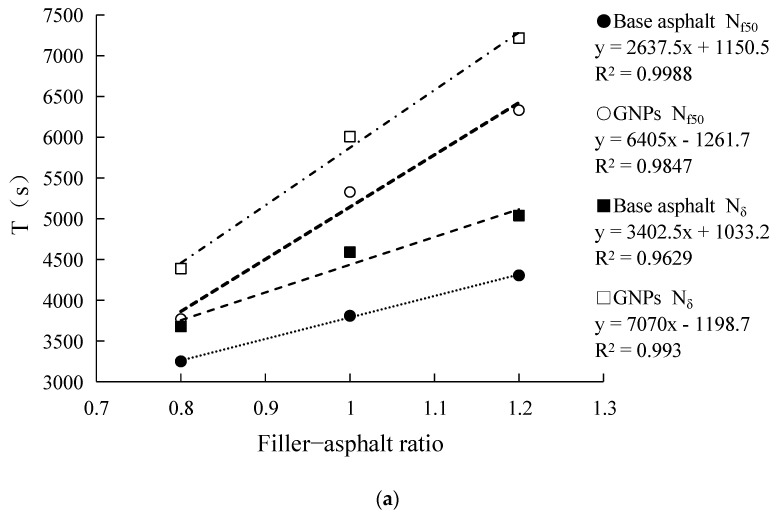

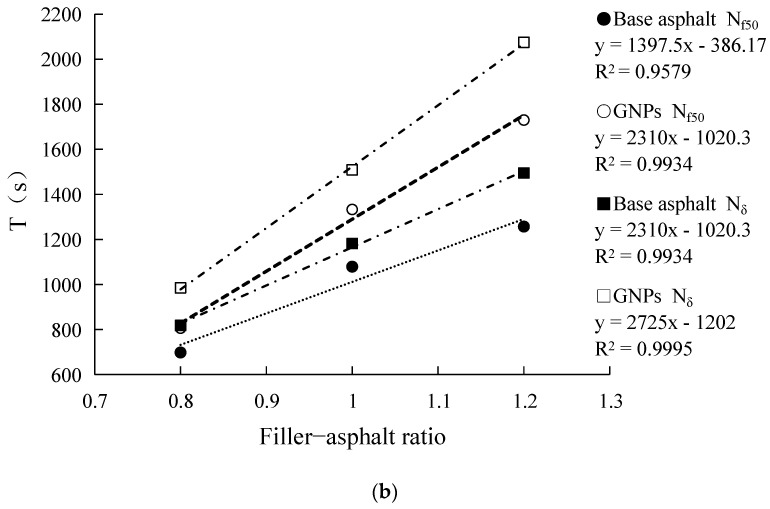
Asphalt mastics fatigue life filler−asphalt ratio curve. (**a**) Asphalt mastics fatigue life filler−asphalt ratio curve (stress 0.15 MPa), (**b**) Asphalt mastics fatigue life filler−asphalt ratio curve (stress 0.2 MPa).

According to the results in Table 6, the fatigue life curve of asphalt mastics is drawn as shown in Figure 2. It can be seen from the fitting equation in the figure that the correlation coefficient R^2^ is above 0.95, which indicates that there is a good linear correlation between filler–asphalt ratio and fatigue life.

#### 3.1.2. Effect of Graphene Nanoplatelets (GNPs) on Fatigue Properties of Asphalt Mastics

**Figure 3:**
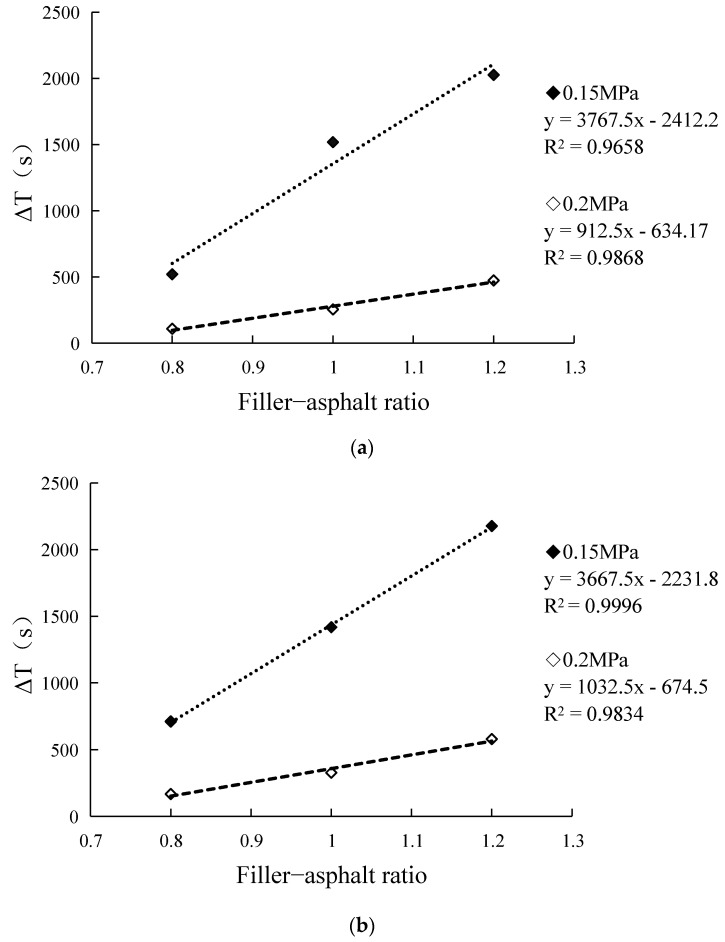
Growth value of fatigue life of GNPs modified asphalt mastics filler−asphalt ratio curve. (**a**) Growth value of fatigue life of GNPs-modified asphalt mastics filler−asphalt ratio curve (N_f50_). (**b**) Growth value of fatigue life of GNPs-modified asphalt mastics filler−asphalt ratio curve (N_δ_).

Based on the results in Table 6, the growth value (ΔT) and growth rate (ω) of fatigue life of GNPs modified asphalt mastics relative to the base asphalt mastics were calculated for each filler–asphalt ratio, and the results are shown in Table 7; and the growth value curve of fatigue life of GNPs modified asphalt mastics was drawn, as shown in Figure 3.

From Table 6, under a stress of 0.15 MPa, the average growth rate ($\bar{\omega }$) of fatigue life of GNPs modified asphalt mastics relative to base asphalt mastics was 17.7% at a filler-asphalt ratio of 0.8, 35.4% at 1.0, and 45.2% at 1.2; under a stress of 0.2 MPa, when the filler–asphalt ratio is 0.8, the average growth rate of fatigue life was 17.9%, 25.6% at 1.0 and 38.2% at 1.2.The results show that the addition of graphene can significantly improve the fatigue life of base asphalt mastics.

From Figure 3, it can be seen that the growth values (ΔT) of fatigue life of GNPs modified asphalt mastics increase correspondingly with increasing filler–asphalt ratio, and the correlation coefficients R^2^ are all greater than 0.95, indicating a good linear relationship between the filler–asphalt ratio and the growth values (ΔT). It can also be seen that the slope of the growth values under 0.15 MPa stress is greater than that under 0.2 MPa stress, indicating that the fatigue life growth under low stress is greater than that under high stress.

### 3.2. Analysis of Fatigue Properties Mechanism of GNPs-Modified Asphalt

Load stress fatigue is the accumulation of unrecoverable bond strength attenuation of asphalt materials under repeated load, which eventually leads to fatigue crack damage [1,2]. From this point of view, the fatigue mechanism of GNPs-modified asphalt mastics was analyzed.

For both base asphalt mastics and GNPs modified asphalt mastics, an increase in the filler-to-asphalt ratio increases the fatigue life of both. This is due to the combination of the mineral particles with the asphalt, which are encapsulated in the asphalt, making the asphalt viscous and exhibiting an increase in the complex modulus, providing more energy to resist deformation and delaying the development of fatigue cracks in the asphalt when resisting external shear forces [27].

There are three reasons why GNPs-modified asphalt mastics have longer fatigue life than the base asphalt mastics:

(1) Because GNPs is interwoven with asphalt, it has an interlayer adsorption effect, which makes the cohesion of asphalt stronger, enhances the toughness of asphalt, thus increasing the modulus of asphalt. When subjected to external shear forces, due to the interlayer adsorption effect of GNPs, a part of the shear stress will be buffered [8,28].
(2) The excellent lubricating performance of GNPs; because the lubricating effect of GNPs between particles in mastics will appear in the process of cyclic shearing [3], the relative position between particles will change, and the particles that were originally in close contact will be at a certain distance. This reduces the damage between the asphalt molecules and the mineral powder particles between the asphalt molecules themselves, and between the mineral powder particles themselves due to shear friction.
(3) The super thermal conductivity of GNPs can transfer heat [4,5], which reduces the heat concentration in asphalt to a certain extent, thus reducing the accelerated damage of asphalt caused by heat generated by friction.

As filler–asphalt ratio increases, the fatigue life growth rate of GNPs modified asphalt also increases correspondingly, because as the increase of mineral powder ratio, the proportion of asphalt decreases, the internal friction base of asphalt increases relatively, and the lubrication effect of GNPs on reducing shear friction is more prominent.

**Figure 4:**
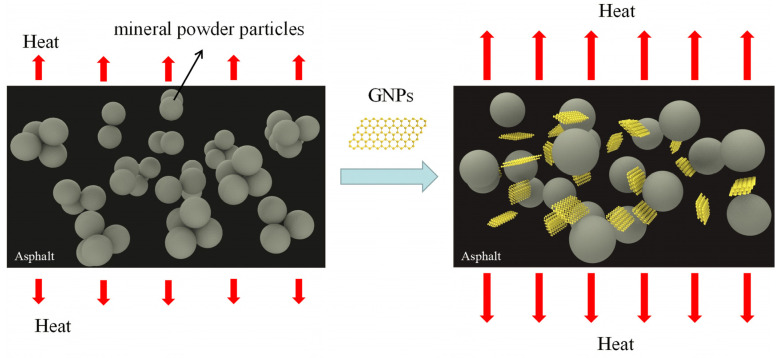
Anti-fatigue mechanism of GNPs-modified asphalt mastics.

The anti-fatigue mechanism of GNPs-modified asphalt mastics is shown in Figure 4.

## 4. Conclusions

In this paper, GNPs-modified asphalt mastics and base asphalt mastics were prepared, with filler–asphalt ratios of 0.8, 1.0 and 1.2, respectively. A dynamic shear rheometer (DSR) was used to perform time-sweep tests on the asphalt mastics with controlled stresses of 0.15 MPa and 0.2 MPa, and the fatigue life of the asphalt mastics was measured under repeated shear loading. The following conclusions were obtained based on the fatigue results analysis:

(1) In the range of 0.8~1.2 filler–asphalt ratio, the increase of mineral powder can improve the fatigue life of asphalt mastics, and the filler–asphalt ratio has a good linear correlation with fatigue life.
(2) GNPs have a significant improvement on the fatigue life of the base asphalt mastics. Under the stress of 0.15 MPa, the average growth rate is 17.7% when the filler–asphalt ratio is 0.8, 35.4% when the filler–asphalt ratio is 1.0 and 45.2% when the filler–asphalt ratio is 1.2; Under the stress of 0.2 MPa, the average growth rate is 17.9% when filler–asphalt ratio is 0.8, 25.6% when filler–asphalt ratio is 1.0 and 38.2% when filler–asphalt ratio is 1.2.
(3) With the increase of filler–asphalt ratio, the fatigue life growth values (ΔT) of GNPs modified asphalt mastics also increase correspondingly, and the correlation coefficients R^2^ is greater than 0.95, which shows that the growth value has a good linear relationship with the filler–asphalt ratio.
(4) On one hand, the fatigue modification mechanism of asphalt mastics modified by GNPs is the interaction between GNPs and asphalt; on the other hand, it is the result of the lubricity and thermal conductivity of graphene itself.

In general, the fatigue resistance of GNPs-modified asphalt mastics is better than that of base asphalt mastics. In the range of commonly used filler–asphalt ratios (0.8–1.2), the higher the filler–asphalt ratio, the more significant the performance of GNPs in improving fatigue life. This research has practical application value to the highway construction industry.

## Figures and Tables

**Table 1:** No. 70 A grade asphalt performance index.

Property		Standard Value	Measured Value	Standard Method
Penetration 25 °C (0.1 mm)		60~80	67.7	T0604
Ductility (10 °C) (cm)		≥25	>100	T0605
Softening point (°C)		≥46	49	T0606
Density (15 °C) (g/cm^3^)		-	1.021	T0603
Rotating Thin Film Oven Test (163 °C, 85 min)	Loss of quality (%)	±0.8	−0.06	T0609
	Penetration ratio (%)	≥61	68.6	T0609 T0604
	Ductility (10 °C) (cm)	≥6	50	T0609 T0605

**Table 2:** Graphene performance indicators.

Carbon Content (%)	Layers	Single Layer Rate	Bulk Density (g/mL)	Moisture Content (%)	Diameter (μm)	Radius-Thickness Ratio
≥98	1–3	≥80%	0.01~0.02	<2.0%	4~7	Average 8500

**Table 3:** Performance index of mineral powder.

Property		Standard Value	Measured Value	Standard Method
Apparent density (t/m^3^)		≥2.50	2.77	T0352
Moisture content (%)		≤1	0	T0103
Particle-size	<0.6 mm (%)	100	100	T0351
	<0.15 mm (%)	90~100	100	
	<0.075 mm (%)	70~100	97.0	
Appearance		No agglomerates	No agglomerates	-
Hydrophilic coefficient		<1	0.6	T0353

**Table 4:** Table of test samples.

No.	Filler–Asphalt Ratio	Types of Asphalt Mastic
1	0.8	Asphalt Mastics
2	1.0	
3	1.2	
4	0.8	GNPs + Asphalt Mastics
5	1.0	
6	1.2	

**Table 5:** Fatigue test parameters.

Parameter	Parameter Value
Stress level (MPa)	0.15; 0.2
Testing temperature (°C)	25
Loading frequency (Hz)	10
Sample diameter (mm)	8
Sample thickness (mm)	2

**Table 6:** Results of fatigue life of asphalt mastics.

Stress Level (MPa)	Filler–Asphalt Ratio	T (s)											
		N_f50_						N_δ_					
		Asphalt Mastics			GNPs + Asphalt Mastics			Asphalt Mastics			GNPs + Asphalt Mastics		
		Samples	σ	Average	Samples	σ	Average	Samples	σ	Average	Samples	σ	Average
0.15	0.8	3221	64	3250	3712	58	3770	3630	91	3678	4329	43	4389
		3190			3850			3599			4423		
		3339			3748			3805			4415		
	1	3708	74	3809	5405	92	5328	4521	50	4590	5928	57	6008
		3890			5380			4610			6052		
		3809			5199			4639			6044		
	1.2	4233	77	4305	6438	77	6332	5080	91	5039	7308	76	7217
		4412			6302			5124			7121		
		4270			6256			4913			7222		
0.2	0.8	674	17	698	819	10	806	806	11	818	1035	36	985
		711			795			832			968		
		709			804			816			952		
	1	1112	35	1079	1346	28	1333	1159	23	1182	1531	67	1509
		1095			1359			1213			1578		
		1030			1294			1174			1418		
	1.2	1198	44	1257	1733	30	1730	1412	75	1495	2066	35	2075
		1305			1692			1480			2122		
		1268			1765			1593			2037		

**Table 7:** Fatigue life growth value (ΔT) and growth rate (ω) of graphene modified asphalt mastics.

Stress Level (MPa)	Filler–Asphalt Ratio	Evaluating Indicator				$\bar{\omega }\;(\%)$
		N_f50_		N_δ_		
		ΔT (s)	ω_1_ (%)	ΔT (s)	ω_2_ (%)	
0.15	0.8	520	16.0	711	19.3	17.7
	1	1519	39.9	1418	30.9	35.4
	1.2	2027	47.1	2178	43.2	45.2
0.2	0.8	108	15.5	167	20.4	17.9
	1	254	23.5	327	27.7	25.6
	1.2	473	37.6	580	38.8	38.2

## Data Availability

All data is contained within the article.
